# Attitudes of medical students in Lahore, Pakistan towards the doctor–patient relationship

**DOI:** 10.7717/peerj.1050

**Published:** 2015-06-30

**Authors:** Waqas Ahmad, Edward Krupat, Yumna Asma, Noor-E- Fatima, Rayan Attique, Umar Mahmood, Ahmed Waqas

**Affiliations:** 1 CMH Lahore Medical College and Institute of Dentistry, Lahore Cantt, Pakistan; 2 Center for Evaluation, Harvard Medical School, Boston, MA, United States of America

**Keywords:** Doctor–patient relationship, PPOS, Patient-centred medical practice, Patient-centred curriculum, Medical student, Pakistan

## Abstract

**Background.** A good doctor–patient relationship is the centre stone of modern medicine. Patients are getting increasingly aware about exercising their autonomy and thus modern medicine cannot deliver all its advances to the patients if a good doctor–patient relationship is not established. We initiated this study with the aim to assess the leaning of medical students, who are the future physicians, towards either a doctor-centered or a patient-centered care, and to explore the effects of personal attributes on care such as gender, academic year, etc.

**Materials & Methods.** A cross-sectional study was conducted between July and Sep 2013. CMH Lahore Medical and Dental College Ethical Review Committee approved the study questionnaire. The study population consisted of 1,181 medical students in years 1–5 from two medical colleges. The English version of Patient Practitioner Orientation Scale (PPOS) was used to assess attitudes of medical students towards doctor–patient relationship. PPOS yields a mean score range of 1–6, where 1 signifies tendency towards a doctor centered relationship and 6 signifies patient-centered relationship. The relationship between PPOS scores and individual characteristics like gender, academic year etc. were examined by multiple regression.

**Results.** A total of 783 students formed the final sample (response rate = 92%). Mean PPOS score of the entire sample was 3.40 (± .49 S.D.). Mean *sharing* sub-scale score was 3.18 (± 0.62 S.D. Mean *caring* sub-scale score was 3.63 (± 0.56 S.D.). Characteristics associated with most patient-centered attitudes were advanced academic year, having a clinical rotation, foreign background and studying in a private college. Gender, having doctor parents, relationship and residence status had no bearing on the attitudes (*p* > 0.05).

**Conclusion.** Despite ongoing debate and the emphasis on a patient-centered curriculum, our study suggests that the current curriculum and its teachings are not producing the results they are designed to achieve. Students should be adequately exposed to the patients from the beginning of their medical education in clinical settings which are more sympathetic to a patient-centered care.

## Introduction

A good doctor–patient relationship is the center-stone of modern medicine. The stronger the relationship, the better the patient’s compliance to the treatment (Choi, Kim & Park, 2004), along with better disease outcomes and patient satisfaction (Heisler et al., 2005; Mallinger, Griggs & Shields, 2005). Just like a weak link in a chain, the doctor–patient relationship is under the most strain when kept vertical (doctor-centered or paternalistic), which doesn’t allow the patient any control over the flow of information or treatment. On the other hand, when it is kept horizontal (patient-centered or egalitarian) the patient is encouraged to play the role of a partner (Campbell & McGauley, 2005) and takes greater responsibility for his own health (Kaba & Sooriakumaran, 2007). It benefits doctors by decreasing the incidence of complaints and litigation (Fallowfield, 2008) and enables them to work at an optimum level to attain the four prima facie maxims (beneficence, non-maleficence, respect for autonomy and justice) (Tor, 2001) of modern medicine.

Patients in the modern era are becoming more and more autonomous (Shankar & Piryani, 2009), an entity seldom considered in the past but modern medicine cannot advance without incorporating this essential ethical necessity (Tor, 2001). Patients question the doctors’ decisions and expect satisfactory answers. This emphasizes the importance of good communication skills in building good doctor patient relationship. Studies have shown that good communication skills can be achieved by structured training, which runs contrary to past beliefs that good communication is an intrinsic quality of a doctor and cannot be taught (Smith et al., 2000).

It is very logical to assess the attitudes of future physicians towards the doctor–patient relationship, which is the very foundation of modern medicine. A growing body of research has demonstrated that medical students around the globe show a wide difference in their attitudes towards the doctor–patient relationship. Researchers have used a valid and reliable scale called the Patient Practitioner Orientation Scale (PPOS) (Haidet et al., 2002) to measure this attitude in countries like Nepal (Shankar et al., 2006), Korea (Choi, Kim & Park, 2004) and Greece (Tsimtsiou et al., 2005). Medical students in Brazil hold highly positive beliefs about patient-centered care (PPOS score of 4.66 ± 0.44 S.D.) (Ribeiro, Krupat & Amaral, 2007), followed closely by American medical students (PPOS score is 4.57 ± 0.48 S.D.) (Haidet et al., 2002).

As indicated by a study in Nepal (PPOS score of 3.71 ± 0.48 S.D.), medical students in Asia have a tendency towards Doctor Centered care (Haidet et al., 2002; Shankar et al., 2006), which is associated with decreased patient satisfaction (Krupat et al., 2000) in many of the countries where this relationship has been studied.

The paucity of knowledge on dynamics of doctor–patient relationship in Pakistani medical schools warranted this study which has been designed with two aims: (1) to assess the leaning of Pakistani medical students towards either a doctor or patient-centered care; (2) to analyze associations of demographic characterstics, year of study and student–patient interaction with patient-centered care as assessed with PPOS.

## Materials & Methods

### Study sample

A descriptive, cross-sectional study design and convenience (non-probability) sampling technique was employed. In Pakistan, undergraduate medical education lasts 5 years; this includes 2 pre-clinical years and 3 clinical years. Students from the 1st and 2nd academic year (pre-clinical years) do not have a clinical rotation in their curriculum. The dominant form of teaching in the medical colleges across the country is conventional, consisting of didactic lecture techniques, non-problem-based learning (PBL) teaching methods, long lectures, tutorials and practical tasks (Waqas et al., 2015). The attitudes of medical students of academic year 1 to 5 from two medical colleges, a government college (Allama Iqbal Medical College) and a private college (CMH Lahore Medical College), were assessed for their attitude towards doctor–patient relationships between July 2013 and Sep 2013. A standardized questionnaire with the English version of PPOS and a series of demographic questions was used. Forms were distributed to 1,274 students (858 in govt. and 416 in private) out of which 1,181 responded (collective response rate 92% (91% and 94.2% respectively)). The total number of students in Allama Iqbal Medical College, Lahore is 1,650, and 650 in CMH Lahore Medical College.

### Instrument

The doctor–patient relationship was assessed by using a reliable assessment tool called the Patient Practitioner Orientation Scale (PPOS) (Haidet et al., 2002). The PPOS contains 18 items and uses a Likert-scale format to measure the respondent’s leaning towards a doctor-centered or a patient-centered belief. Each item has 6 possible responses ranging from 1 (strongly agree) to 6 (strongly disagree). The scale has two subscales which measure two domains of doctor–patient relationship: Sharing and Caring. Sharing refers to an individual’s belief that a patient should share the power, control and flow of information equally with their doctor. Caring refers to an individual’s belief that a patient should be as a whole and with good emotional rapport rather than as a condition or disease. Both sub-scales have 9 items each. PPOS yields a mean score range of 1–6, where 1 signifies tendency towards a doctor centered relationship and 6 signifies patient-centered relationship.

### Statistical analysis

SPSS Inc., (Chicago, Illinois, USA) version 21 software was used for analysis. Descriptive statistics and frequencies were calculated for subscale scores on PPOS and demographic variables, respectively. Multiple regression analysis (backward method) was run to predict PPOS scores, Sharing and Caring subscale scores from gender, age, study year, rotation (outpatient department, inpatient department and not applicable) and nationality (Pakistani/overseas). Students having different residence (off campus/in campus) and relationship status were hypothesized to have different attitudes towards doctor–patient relationship because of their exposure to different psycho-social stressors and hence included in the regression analysis. The assumptions of linearity, independence of errors, homoscedasticity, unusual points and normality of residuals were assessed.

### Ethics statement

CMH Lahore Medical and Dental College Ethical Review Committee approved the study questionnaire. Permission was also granted for data collection by Dean of Allama Iqbal Medical College, Lahore.

## Results

Students from academic years 1–5 of both colleges participated in this study (*N* = 1,181). Out of 1,181 forms, 398 were discarded due to incomplete demographics and more than 3 missing responses in PPOS (final sample *N* = 783). There were 226 (28.9%) males and 557 (71.1%) female students. The sample distribution by gender, college, academic year etc. is shown in Table 1. The mean PPOS score of the entire sample was 3.40 (±0.49 S.D.). Mean Sharing sub-scale score was 3.18 (±0.62 S.D.). Mean caring sub-scale score was 3.63 (±0.56 S.D.). Multiple regression analysis yielded significant models for mean PPOS scores, Sharing and Caring scores (Table 2). Mean PPOS scores were positively associated with students from privately financed medical college and foreign background. Students rotating in inpatient or outpatient departments (OPDs) scored significantly higher on PPOS scale than those who were not yet rotating in a clinical setting. Similar trends were observed in Sharing and Caring domains. Students having a foreign background, currently in a higher academic year and studying in a privately financed medical college were associated with higher scores on the Sharing sub-scale. Scores on the Caring sub-scale were positively associated with foreign background and rotation in outpatient (OPDs) an inpatient departments. Residence, relationship status and having parents who are doctors had no bearing on doctor–patient relationships (*p* > 0.05).

**Table 1:** Demographic characteristics of the students (*N* = 783).

	*n* (%)
**College**	
Allama Iqbal Medical College	509 (65%)
CMH Lahore Medical College	274 (35%)
**Gender**	
Male	226 (28.9%)
Female	557 (71.1%)
**Academic year**	
1st year	173 (22.1%)
2nd year	145 (18.5%)
3rd year	177 (22.6%)
4th year	183 (23.4%)
5th year	105 (13.4%)
**Country of origin**	
Pakistan	750 (95.8)
Foreign	33 (4.2%)
**Doctor parents**	
Yes	197 (25.2%)
No	586 (74.8%)
**Residence**	
On campus	416 (53.1%)
Off campus	367 (46.9%)
**Relationship**	
Single	722 (92.2%)
Married	21 (2.7%)
Having a boyfriend/girlfriend	40 (5.1%)
**Clinical rotation**	
Outpatient department	63 (8.0%)
Ward	402 (51.2)
Not applicable^a^	318 (40.7)

**Notes.**

a Not applicable refers to the students from 1st and 2nd academic year who do not have a clinical rotation in their curriculum.

**Table 2:** Multiple regression model for mean PPOS and sub-scale scores (*N* = 783).

Predictors	B	Std. error B	Beta	*P* value
**Mean PPOS scores** (Adj. *R*^2^ = .063, *P* < .001)				
Constant	3.1	.13		<.001
CMHLMC vs. AIMC	−.13	.04	−.12	<.001
Pakistani vs. Foreign	.36	.09	.15	<.001
N/A vs. OPD	.26	.07	.14	<.001
N/A vs. Ward	.12	.04	.12	<.001
Residence (On campus/off campus)	.06	.04	.06	>.05
**Mean Sharing sub-scale scores** (Adj. *R*^2^ = .061, *P* < .001)				
Constant	2.9	.15		<.001
CMHLMC vs. AIMC	−.23	.05	−.18	<.001
Pakistani vs. Foreign	.43	.12	.14	<.001
Study year	.05	.02	.12	<.001
**Mean Caring sub-scale scores** (Adj. *R*^2^ = .028, *P* < .001)				
Constant	3.141	.125		.000
Pakistani vs. Foreign	.296	.099	.105	<.01
N/A vs. OPD	.275	.077	.133	<.001
N/A vs. Ward	.110	.042	.098	<.01
Residence (on campus/off campus)	.072	.040	.064	>.05

**Notes.**

N/A Student of 1st and 2nd academic year who do not have any clinical rotation in their curriculum OPD Out Patient Department AIMC Allama Iqbal Medical College CMHLMC CMH Lahore Medical College PPOS Patient–Practitioner Orientation Scale

## Discussion

Our findings suggest that Pakistani medical students very much believe in “Doctor Knows Best” (Tor, 2001). They scored even lower than their Nepali counterparts (Shankar et al., 2006), except in the Caring domain, making them the most doctor-centered of those samples of medical students in several studies done around the world. Women, who are traditionally associated with patient-centered care and are shown to have a leaning towards it (Haidet et al., 2002), had statistically the same distribution of PPOS scores as males of this sample (*p* > 0.05). This finding, although contradictory to the studies conducted in America and Brazil, is consistent with findings in Nepal (another Asian country) (Haidet et al., 2002; Shankar et al., 2006; Ribeiro, Krupat & Amaral, 2007). This consistency might be due to social, religious and cultural differences present in the two continents i.e., Americas and Asia.

Relationship status had no significant association with mean PPOS scores. Our sample had a very small proportion of students who were involved in a premarital relationship, as premarital relationships in this part of the world are discouraged based on religious and cultural norms. No clear pattern was established in consecutive academic years in terms of mean PPOS scores or caring sub-scale scores. However, Sharing domain scores showed a clear positive association with higher academic years, which suggests that students, as they go into higher academic years, become more aware about the rights of the patients and are willing to share the power of treatment choices with them. This finding is similar to the study done in Brazil (Ribeiro, Krupat & Amaral, 2007) but contradictory to the ones done in USA where good patient-centred care is associated with early academic years (Haidet et al., 2002). Attributes associated with leaning towards patient-centred care were: studying in a privately financed medical college, having a foreign background, and rotating in outpatient or inpatient departments as opposed to those who were not currently rotating in a clinical setting. These attributes were consistent throughout the mean PPOS scores and the Sharing sub-scale. Privately-financed medical colleges have a better teacher to student ratio with a lesser patient load in attached private hospitals as opposed to government-financed colleges. This might explain the higher scores of students from private medical schools. Another interesting finding in our study was that students who had a clinical rotation (students in clinical years), either in outpatient or inpatient departments, scored better than those who did not have a rotation (students in pre-clinical years). These finding clearly divide our sample into the ones who just see patient on pages of books while the others who interact with them and see them as a whole. It also illustrates the importance of patient interactions and necessitates that the student–patient interaction to begin at an early stage of medical training. Students who rotated in an inpatient setting showed a stronger positive association with mean scores on PPOS than those who rotated in an outpatient setting, which could be due to a continuous flow of patients in outpatient setting who have a very brief interaction with doctor/students as opposed to patients in wards who stay there for long durations and offer a better chance for students to get to know them and see them as a whole person rather than a disease. Better performance by foreign students might be due to not sharing the Asian culture, which is associated with doctor-centered care (Haidet et al., 2002).

Medical students join this profession of medicine to heal patients (Lloyd-Williams & Dogra, 2004) but instead are taught to only heal the disease. The present system of medical education does not necessitate the development of characteristics like good communication skills, etc., which are necessary for good patient-centered care. The pressures they are exposed to (academic, psycho-social and health related) further retard their growth into a patient-centered practitioner (Waqas et al., 2015).

Although this does not mean that medical students cannot develop these skills after leaving medical school, it would be much more beneficial to the patients and healthcare system if they were taught to focus on the patient as a whole sooner than later in their medical career. Another reason for medical students to be more doctor-centered could be due to the teaching style of the practicing doctors who teach them. The environment in which clinicians teach is not always conducive to the high ideals of the doctor–patient relationship that the students are taught in lecture theatres (Grilo et al., 2014). This opinion is enforced by Humayun et al. (2008), who found that Pakistani doctors did not take informed consent from more than 71% patients and provided adequate confidentiality to less than 24% of their patients. Informed consent is defined as “patients’ autonomy in decisions and right to complete information” and confidentiality entails the right of the patient to informational privacy (Humayun et al., 2008). When medical students are taught in such a doctor-centered environment, it is natural for them to embody such practices; when students realizes that doctors who do not following the prima facie maxims (Tor, 2001) are still able to have a very healthy practice, then they wonder if formalities like consent or confidentiality even matter in the real world. Doctors in the government-owned hospitals did not take consent from more than 90% patients, and provided adequate confidentiality to less than 11% of their patients (Humayun et al., 2008). The teaching provided by these doctors could explain our finding that medical students from government-owned medical schools scored lower on the PPOS than those of private medical schools (*p* < 0.05).

Shaikh et al. (2004) have reported the prevalence of stress to be 90% in Pakistani medical students. Further studies have shown that psychological stress can cause poor attitudes towards the chronically ill, decreased empathy and high levels of cynicism (Crandall, Volk & Loemker, 1993) which together amount to a less favorable patient care. Students who experience these stresses seldom seek help because of the stigma surrounding psychiatric illnesses (Waqas et al., 2014). For this reason, medical educators should make it mandatory to see the prevalence of such psychiatric illnesses/stressors during the course of medical education and should take prompt actions to protect students from their harmful effects.

## Conclusion

If medical schools want to develop physicians who treat the patient as a whole, then medical educators would have to initiate new programs to develop the essential characteristics (like good communication skills, empathy etc.) necessary to achieve the *prima facie maxims* of modern medicine. Students should be adequately exposed to patients from the beginning of their graduate program and in clinical settings which are more favorable to a patient-centered care. Continuous monitoring of the students should be done to identify and mend the factors which push them away from a patient-centered caring attitude, and patient-centered role models should be sought for students to observe and follow. Most of what is learned during the graduate program is through a “Hidden Curriculum,” which is a set of influences functioning at the level of organizational structure and culture (Hafferty, 1998). This is mostly true for Pakistan since its biggest medical university (UHS) introduced Behavioral Sciences as an integral part of the curriculum in 2007, which is yet to produce its effects in medical practice of Pakistan.

### Limitations and suggestions for future research

Despite our efforts to completely explore the attitude of medical students towards doctor–patient relationships, we strongly believe that additional factors should be incorporated into further research done in the future in this domain.

1. Academic staff from the respective colleges and hospitals were not included in this study, which could have helped in measuring the extent of the problem.
2. We conducted our study in just one city. Further studies should include a broader sample comprised of medical students from all four provinces and all religious and ethnic groups to see if these factors have any effect on Patient-Centered Care.
3. The cross-sectional design of this study limits inferences about causality and temporality.
4. Since PPOS only measures the orientation and not the behavior of medical students towards Patient-Centered care, future researchers should include means to see the behavior of medical students toward this entity.

## Supplemental Information

10.7717/peerj.1050/supp-1 Supplemental Information 1 SPSS Data Set
